# Do you really want to deactivate your sacral neuromodulation device during pregnancy? A single center case series

**DOI:** 10.1007/s00192-020-04594-w

**Published:** 2020-11-11

**Authors:** Marco Agnello, Mario Vottero, Paola Bertapelle

**Affiliations:** 1 Università degli Studi di Torino-Scuola di Medicina-Dipartimento di Scienze Chirurgiche, Urologia U, A.O.U. Città della Salute e della Scienza di Torino, Presidio Molinette (Corso Bramante 88), 10126 Torino, Italy; 2 SC Neuro-Urologia, A.O.U. Città della Salute e della Scienza, Torino, Italy

**Keywords:** Delivery, Electrical stimulation, Peripheral nerve stimulation, Pregnancy, Sacral neuromodulation, Safety

## Abstract

**Introduction and hypothesis:**

The main objective of the study is to assess the efficacy and safety of sacral neuromodulation (SNM) during pregnancy.

**Methods:**

We retrospectively enrolled patients who underwent SNM implantation in our center and subsequently became pregnant. The indication for SNM, timing of device de-activation (if performed), course of pregnancy and urological complications, duration of labor, childbirth term, delivery mode, congenital abnormalities and SNM dysfunctions after delivery were recorded.

**Results:**

Fourteen pregnancies were recorded among 11 women undergoing SNM. Indications for device implantation were urinary retention (7 cases) and dysfunctional voiding (4 cases). Two patients carried on two and three pregnancies, respectively, with the device turned off since the first trimester. They both had to return to self-catheterization and developed recurring urinary tract infections. No major urological complications were recorded among the remaining nine women that kept the device on during pregnancy. A cesarean section was performed in four cases for obstetric reasons, and in seven cases it was planned by the urologist and gynecologist to avoid lead damage/displacement. Three pregnancies resulted in a vaginal delivery, and no association with term of delivery or duration of labor was observed. No congenital abnormalities related to SNM or lead displacement are reported, and only one patient required device removal because of significant loss of efficacy after childbirth.

**Conclusions:**

The use of SNM during pregnancy appears to be safe, without morbidity for the fetus. Moreover, risks associated with switching the device off may be greater than benefits and justify maintaining the electrical stimulation throughout pregnancy.

## Introduction

Safety and effectiveness of sacral neuromodulation (SNM) during pregnancy and the impact of delivery on SMN function have not yet been fully established. Few animal models have been developed to study the effect of SMN on the endometrium [1], and most case reports and case series on humans agree with the parent company on turning the device off, because of its largely unknown effects on pregnancy and the fetus.

On the other hand, keeping the device deactivated during pregnancy may increase the risk of urinary retention (UR) and urinary tract infections (UTI) and worsen urinary symptoms, especially if the indication for SNM implantation was non-obstructive UR or dysfunctional voiding. In such cases, the choice of indwelling catheter or intermittent self-catheterization may even worsen the course of pregnancy.

Furthermore, increase of abdominal girth and mass effect [2], hormonal changes, pushing efforts during delivery, anesthetics and surgical procedures [3] may interfere with device function.

Our retrospective study has two main objectives. The first is to evaluate whether the beneficial effect of SMN on urinary symptoms is maintained throughout pregnancy and lost after device deactivation. The second objective is to assess the safety of chronic stimulation during pregnancy in terms of fetal complications. Secondary outcomes are the effects of SNM on childbirth term and labor duration, the impact of SNM on delivery mode decision making and the effects of delivery on SNM function.

## Materials and methods

We retrospectively included patients who underwent SNM implantation in our center and subsequently carried on a pregnancy. The first considered implanted device and pregnancy dated back to 1997 and 2000, respectively. The last pregnancy was carried on in 2020, in a patient who underwent an SNM implant in March 2011. All implants were performed by the same qualified team of two specialized urologists using a Medtronic® quadripolar lead and Interstim® implantable pulse generator (IPG). The indication for SNM, status of the device during pregnancy (and timing of de-activation and re-activation, if performed), course of pregnancy and urological complications, duration of labor, childbirth term, delivery mode, type of intraoperative anesthesia (if present), congenital abnormalities and device status after delivery were recorded. All data were collected after obtaining informed written consent as well as our Institutional Review Board approval (protocol no. 0056532) using the patient’s medical records and phone interviews.

## Results

Fourteen pregnancies were recorded among 11 women undergoing SNM. Mean age at implant was 28.6 years. Reasons for device implantation were dysfunctional voiding in four cases and UR in seven cases. In six out of seven cases, UR was idiopathic. In the last case, UR followed a pelvic surgery to drain a uterine hematoma after a complicated vaginal delivery. Table 1 shows the patients’ characteristics.

**Table 1:** Patient characteristics. Patients are sorted by date of device implant. Patient 1 and 2 carried on two and three pregnancies, respectively (indicated by subsequent letters on patient ID). Dysfunctional voiding was considered when all the following parameters were observed: a maximum flow rate < 15 ml/s; a maintained detrusor contraction (> 25 cmH_2_O) in the presence of incomplete bladder emptying (post-void volume > 100 ml and voided volume > 200 ml); an increase in EUS EMG activity during micturition. OR = operating room. SNM = sacral neuromodulation. UR = urinary retention. UTIs = urinary tract infections

Patient ID	Implant reason	Implant date	Age at implant	Implant technique		Time from implant to delivery (months)	Device shutdown during pregnancy	Urological complications during pregnancy	Pain at lead/battery site	Battery site	Abortion	Childbirth term	Labor duration (hours)	Type of delivery	Anesthetic during labor and delivery	Cesarean reason (if performed)	Congenital abnormalities	SNM dysfunction after delivery	SNM removal after pregnancy
1a	Idiopathic UR	01/03/1997	21	Open surgery		42	**Yes, from beginning of pregnancy to the end**	Worsening of bladder emptying and need for self-catheterization. Loss of spontaneous micturition with device turned off. Recurrent UTIs treated with antibiotics and an episode of pyelonephritis with hospitalization at month 7	No	Gluteal region	No	Pre-term	10	Vaginal	None		No	Worsening of urinary symptoms after delivery and device re-activation, with dysuria and urgency. Better after new parameter setting	No
1b	Idiopathic UR	01/03/1997	21	Open surgery		75	**Yes, from beginning of pregnancy to the end**	Worsening of bladder emptying and need for self-catheterization. Loss of spontaneous micturition with device turned off. Recurrent UTIs treated with antibiotics	No	Gluteal region	No	Post-term	6	Vaginal	None		No	Patient experienced a new worsening of urinary symptoms after delivery and device re-activation, with dysuria and urgency. Progressive loss of spontaneous micturition	**Yes**
2a	Idiopathic UR	01/01/1999	29	Open surgery		73	**Yes, from beginning of pregnancy to the end**	Worsening of bladder emptying and need for self-catheterization. Loss of spontaneous micturition with device turned off. Recurrent UTIs treated with antibiotics. Indwelling catheter the last 2 months of pregnancy	No	Abdominal region	No	Term	Planned cesarean	Cesarean	Regional anesthesia	Gynecologist and urologist decision to avoid abdominal pushing and lead displacement/damage, moreover considering great efficacy of therapy and difficulty in the previous open-surgery implant	No	After device re-activation, patients started to empty the bladder again with spontaneous micturition	No
2b	Idiopathic UR	01/01/1999	29	Open surgery		115	**Yes, from beginning of pregnancy to the end**	None	No	Abdominal region	No	Term	Planned cesarean	Cesarean	Total (allergy in OR)	Gynecologist and urologist decision to avoid abdominal pushing and lead displacement/damage, moreover considering great efficacy of therapy and difficulty in the previous open-surgery implant	No	None	No
2c	Idiopathic UR	01/01/1999	29	Open surgery		149	**Yes, from beginning of pregnancy to the end**	None	No	Abdominal region	No	Term	Planned cesarean	Cesarean	Regional anesthesia	Gynecologist and urologist decision to avoid abdominal pushing and lead displacement/damage, moreover considering great efficacy of therapy and difficulty in the previous open-surgery implant	Psychomotor developmental delay (diagnosis after 2 years)	None	No
3	Idiopathic UR	03/07/2001	24	Open surgery		19	**Temporary** de-activation in 3rd trimester. Re-activation after a few weeks	Device shutdown in 3rd trimester to spare battery life. During deactivation period, an episode of UR occurred and an indwelling catheter was temporarily placed. Device was re-activated after a few weeks and kept on during all the rest of pregnancy	No	Abdominal region	No	Pre-term: planned cesarean, anticipated for early contractions	Planned cesarean	Cesarean	Regional anesthesia	Obstetric/surgical reason (previous abdominal surgeries). Gynecologist asked for urologist in OR to avoid stimulator damage	No	None	No
4	Dysfunctional voiding	24/07/2002	31	Percutaneous		39	**Temporary** de-activation in 2nd trimester. Re-activation after a few weeks	Device shutdown in 2nd trimester to spare battery life. After deactivation, an episode of UR occurred. Device was activated again and kept on during all the rest of pregnancy	No	Gluteal region	No	Pre-term	Planned cesarean	Cesarean	Regional anesthesia	Obstetric/surgical reason (breech)	No	None	No
5	Idiopathic UR	07/05/2007	33	Percutaneous		149	No	UTIs, treated with antibiotic. No relation with SNM or worsening of bladder emptying	Increased perception of paresthesias typical of neuromodulation during fetus movements, but no pain or need to decrease voltage	Gluteal region	No	Term	Planned cesarean	Cesarean	Regional anesthesia	Obstetric/surgical reason (induced 2 weeks before for gestational hypertension)	No	UR after delivery, which lasted 2 weeks (need for self-catheterization). Device always functioning during this period. Further reappearance of spontaneous micturition	No
6	Dysfunctional voiding	05/01/2011	29	Percutaneous		10	No	None	No	Gluteal region	No	Term	Planned cesarean	Cesarean	Regional anesthesia	To avoid abdominal pushing and lead trauma (according to gynecologist and urologist)	No	None	No
7	Dysfunctional voiding	27/11/2011	29	Percutaneous		99	No	None	No	Gluteal region	No	Term	Planned cesarean	Cesarean	Regional anesthesia	To avoid abdominal pushing and lead trauma (according to gynecologist and urologist)	No	None	No
8	Idiopathic UR	11/04/2012	17	Percutaneous		72	No	UTIs treated with antibiotic. No relation with SNM or worsening of bladder emptying	No	Gluteal region	No	Term	12	Vaginal	None		No	None	No
9	Idiopathic UR	26/06/2013	29	Percutaneous		46	No	None	No	Gluteal region	No	Term	Planned cesarean	Cesarean	Regional anesthesia	To avoid abdominal pushing and lead trauma (according to gynecologist and urologist)	No	None	No
10	Dysfunctional voiding	05/06/2015	38	Percutaneous		14	No	None	No	Gluteal region	No	Term	Planned cesarean	Cesarean	Regional anesthesia	Obstetric/surgical reason (previous abdominal surgeries for deep infiltrating endometriosis)	No	None	No
11	UR after vaginal delivery and pelvic surgery to drain uterine hematoma	07/10/2015	35	Percutaneous		49	No	None	No	Gluteal region	No	Pre-term (placental abruption)	Planned cesarean	Cesarean	Regional anesthesia	To avoid abdominal pushing and lead trauma (according to gynecologist and urologist)	No	None	No

Patient 1 and patient 2—affected by idiopathic UR and with spontaneous micturition after SNM implant—carried on 2 and 3 pregnancies respectively, with the device turned off since the first trimester. Both patients—who underwent an SNM implant before 2000 with an open technique—experienced a loss of spontaneous micturition with the device off, the need to restart self-catheterization during pregnancy and recurrent UTIs; an episode of pyelonephritis with hospitalization in the third trimester also occurred.

In nine the patient's device was kept on during pregnancy.

The device was temporarily switched off in patient 3 and patient 4 in the second and third trimester, respectively, to minimize battery consumption. After turning the device off, a recurrence of urinary retention was reported. Both patients turned the device on after a few weeks and were able to achieve spontaneous voiding. Two patients (patient 5 and patient 8) experienced an episode of uncomplicated lower UTI, treated with antibiotics, with no negative effect on SNM efficacy on bladder emptying.

No other major urological complications during pregnancy occurred.

Patient 5 reported an increased perception of typical SNM perineal paresthesias during movements of the fetus, but did not experience pain or need to decrease the amplitude of electrical stimulation.

SNM was deactivated during labor in all subjects to avoid magnetic or electrical interference between the lead, IPG and surgical devices in the operating room (in the event of a cesarean section or complication of vaginal delivery). It was re-activated 2 days after delivery, and an indwelling catheter was used in the meantime.

A cesarean section (CS) was performed in 11 out of 14 pregnancies.

The indication for CS was exclusively obstetric in patient 4 and patient 5 (gestational hypertension and breech) and secondary to previous pelvic surgery in patient 3 and patient 10. In the remaining seven cases CS was jointly planned by the urologist and gynecologist to avoid lead displacement in case of prolonged and painful delivery.

In ten cases a regional anesthesia was administered, and only one patient underwent general anesthesia because of an adverse drug reaction in the operating room.

All CS were planned and performed at full term, except for two cases (patient 3 and patient 11) where obstetric complications (placental abruption and early contractions) led to a pre-term surgical delivery.

Three pregnancies (patient 1 and patient 8) resulted in a vaginal delivery (pre-term, full term and post term, respectively). Two of these pregnancies were carried on by a patient (patient 1) affected by idiopathic non-obstructive UR who experienced, after the first delivery, a worsening of bladder emptying (post-void residual > 100 ml compared with post-void residual < 50 before delivery). Satisfactory control of symptoms was achieved by SNM parameter modification. Following the second pregnancy, symptoms worsened again, and the patient required SNM removal because of a significant loss of device efficacy, despite correct anatomical positioning confirmed on x-ray.

No lead displacement during or after pregnancy and no congenital abnormalities were observed in any of the subjects. One child was diagnosed with a psychomotor developmental delay at 2 years of age and was the third of three children born to the same mother (patient 2), who had been implanted with SNM prior to her first pregnancy. Furthermore, SNM had been turned off for the entire duration of all the three pregnancies.

## Discussion

Currently available data on the efficacy and safety of sacral neuromodulation during pregnancy are scarce and mainly based on research studies with a low level of evidence. For this reason, the parent company as well as numerous urological and gynecological associations—including the International Urogynaecological Association (IUGA)—suggests keeping implantable pulse generators (IPGs) off during pregnancy, as “the effect of neuromodulation on pregnancy is largely unknown.” A national survey carried out by emailing all the urologists listed in the Interstim National Registry in France showed that deactivation of the device was advised in 18.5% of cases while trying to conceive and during the first trimester in the remaining 81.5% cases [4].

Two systematic reviews are currently available on the topic. Both appear to suggest that no negative effects of SNM on the fetus, mother or device are significantly reported in the literature, although they come to divergent conclusions: while Mahran and colleagues [5] suggest that decisions on SNM management should be individualized after a full discussion of benefits and risks with the patient, Yaiesh [6] more explicitly supports the recommendation of commencing SNM treatment during pregnancy if there is no contraindication to it.

The main concerns and doubts about SNM's effect on pregnancy, which emerge from previous studies and ours, are here reported and analyzed in detail.

### Teratogenic effects on the fetus

SMN can be considered safe during pregnancy if sufficient data show that it does not interfere with fetal development, particularly during the first trimester, when teratogenic effects are most likely to occur. In the literature, no significant congenital anomalies or miscarriages that may be directly related to SNM are reported. Khunda [7] described a miscarriage in a patient previously implanted with SNM. This patient underwent IVF treatment after the SNM device was turned off; therefore, no direct correlation between pregnancy loss and electrical stimulation was identified. El-Khawand [8] reported the case of two newborns from the same mother with a pilonidal sinus cyst and motor tic disorder, respectively. A genetic predisposition may be suggested by the occurrence of congenital abnormalities within pregnancies in the same mother, but data about genetic inheritance of this family are not available [5]. Bernardini [3] described cases of accidental electrical shocks and use of cardioversion to revert arrhythmias in pregnant women and did not find a significant difference in cardiac abnormalities in these groups compared to the average population. Considering the high-voltage exposure to electrocution and shock waves, and the significantly lower voltages of sacral and spinal cord stimulators, the author supports the safety of these devices, but does not deviate from the consensus of switching them off during pregnancy.

In our study, no congenital abnormalities were observed among the women that kept the device on during pregnancy. One case of psychomotor developmental delay was observed in a child born of a woman (patient 2) who had the device deactivated prior to pregnancy.

### Influence of SNM on uterine contractions and labor

Uterine quiescence during pregnancy is mainly maintained by circulating hormones and peptides. At term, the uterus becomes myogenic, similarly to the myocardial muscle, and contracts spontaneously without neural control, because of hormonal and peptidyl changes that facilitate the creation of gap junctions, cell interconnections and current flow.

In animal models, Karsdon [1] demonstrated that direct or indirect electrical stimulation of rat and rabbit myometrium inhibits the typical spontaneous uterine pre-term and at-term gestational contractions, with reduction of 50–80% intra-uterine pressure. This electrical inhibition is effective with a rapid onset and rapid reversal. Govaert [9] analyzed myometrial activity through transvaginal ultrasounds in six women under SNM for fecal incontinence, but obtained conflicting data and concluded that, despite “some effect of SNS on uterine activity,” no guidelines for SNS usage during conception and pregnancy could be recommended.

Although a direct stimulation of the myometrium—as shown by Karsdon—may cause electrical inhibition of uterine contractility, sacral nerve stimulation is not a direct stimulation of the uterus. If we assume, as suggested by Bernardini and colleagues [3], that uterine activation and contraction during labor and delivery are primarily regulated by hormones and peptides, and no correlation between electrical stimulation during pregnancy and changes in levels of systemic hormones and neurotransmitters have been proven yet [1], the hypothesis of a slowing of labor due to SNM is less suggestive. Moreover, an association among SNM, labor and the timing of delivery has never been described.

According to these considerations and despite our low numbers, we did not observe a significant correlation between SNM and term of delivery. Only in patient 3 was the previously planned cesarean delivery moved up by 2 weeks because of the occurrence of early uterine contractions.

### Occurrence of urinary symptoms after turning the device off during pregnancy

A major concern is related to a possible worsening of urinary symptoms, development of UTIs, episodes of urinary retention or need for self-intermittent catheterization or an indwelling catheter when the device is turned off during pregnancy. This especially applies to cases where urinary retention was the indication for SNM implantation and electrical stimulation was effective in facilitating bladder emptying. Considering the lack of evidence of a direct teratogenic effect or influence on delivery by SNM, the question should be raised whether it is appropriate to deactivate the device during pregnancy, exposing both the patient and fetus to the risks mentioned above. The systematic review by Mahran and colleagues [5] reported urological complications or worsening of urinary function only in patients who deactivated the device either before or early during pregnancy: of 18 patients that deactivated the device during pregnancy, 2 requested SNM re-activation for recurrent symptoms of UR and fecal and urinary urgency, respectively. Seven pregnancies were complicated by UTIs, including one case of pyelonephritis that required hospitalization and intravenous antibiotics.

In our study, the indication for an SNM implant was UR in all cases, whether idiopathic or due to pelvic trauma or dysfunctional voiding. The two patients that kept the device switched off for the entire course of their pregnancies (patient 1 and patient 2) had undergone SNM implantation many years ago (1997 and 1999, respectively), when experience with SNM during pregnancy was limited, concerns about the fetus and delivery were prevalent, and no data were available in the literature. In these patients, switching the device off resulted in episodes of UR and recurrent UTIs, treated with self-intermittent catheterization or an indwelling catheter for a certain period; we also experienced a pyelonephritis that required hospitalization at month 7 in patient 1. In the following two cases (patient 3 and patient 4; SNM implant in 2001 and 2002), the device was temporarily switched off in the first and second trimester, respectively, to spare battery life. The onset of an episode of total urinary retention in both cases after turning the device off led to its re-activation after a few weeks.

Based on this experience, the other seven more recent patients carried on their pregnancies, keeping the device on until delivery. Of these patients, one uncomplicated lower UTI appeared in two cases (patient 5 and patient 8), but was successfully treated with antibiotics with no sequalae. No major urological complications were recorded, suggesting a benefit from the SNM device that lasts over pregnancy.

### Problems related to an abdominal and gluteal subcutaneous pocket for IPG

There is weak evidence that abdominal placement of IPGs may lead to technical and biological complications. Wiseman and colleagues [2] reported the case of an IPG repositioning due to pain at the level of abdominal pocket during pregnancy, probably related to a physiological increase in abdominal girth. Moreover, skin ulceration of the abdominal IPG during pregnancy is described in cardiac pacemakers [10]. Abdominal IPG may also be damaged during an emergency CS [3]. We did not report pain, skin thinning or ulceration at the lead or battery site or battery or lead extension damage if a cesarean section was performed in a patient with the stimulator in the abdominal region, although in one case (patient 3) the gynecologist required support from the urologist during surgery. At present, IPG placement in the gluteal pocket is the gold standard technique and should not pose any clinical issues during pregnancy.

### Cesarean section or vaginal delivery?

Another frequent dilemma is whether to offer a CS or a vaginal delivery in this category of patient. The main concerns related to a vaginal delivery relate to increased abdominal pressure during pushing efforts, which may lead to electrode displacement or damage. Accordingly, patients may experience a loss of benefits from chronic stimulation, a worsening of urinary symptoms and the need to re-start or increase the number of daily self-intermittent catheterizations to complete bladder emptying. Removal of a displaced or damaged lead could be remarkably challenging, particularly in cases where the lead has been fixed to the periosteum of the sacral bone using surgical screws through an open procedure (patient 1, 2 and 3 of our series). The more time that passes from the electrode placement, the harder it becomes to remove it because of the tenacious adhesions to the sacrum and surrounding tissues and to place a new lead on the same side (probably the most effective side was tested during electrode placement). Importantly, the risk of prolonged labor and delivery in a hypertonic pelvic floor—not infrequent in dysfunctional voiding—should not be underestimated. On the other hand, CS has often been considered with caution by gynecologists to avoid lead extension or IPG damage, especially when the stimulator is placed in a subcutaneous pocket on the anterior abdominal wall. Some authors suggest performing a CS to avoid lead displacement during labor and delivery [2, 11]; others [7] reported higher rates of device malfunctioning after CS compared to vaginal delivery and advised to “avoid caesarean section delivery for patients with implanted device unless an obstetric indication exists.” However, there is no evidence that a specific delivery mode is associated with an augmented risk of lead displacement and lead migration documented on x-ray after delivery has never been described.

In our population, most pregnancies (11 out of 14) ended with a CS. Excluding the cases with obstetric complications, CS was performed following the instructions of urologists and gynecologists to avoid lead damage or displacement during pushing efforts for the reasons mentioned above. The case of patient 2, where lead placement using the open technique (the gold standard at the time) had been challenging because of difficult sacral anatomy and tissue adhesions, is somehow emblematic. In this case, CS was chosen to avoid lead displacement and the possible need to reposition it.

### Epidural and subarachnoid anesthesia during labor

In two cases of our series anesthesiologists were unsure whether to choose subarachnoid or epidural anesthesia during labor to obtain analgesia. The main concern was about damaging the quadripolar lead during the procedure. Considering the great distance from the typical lumbar access to the subarachnoid/epidural space and third and fourth sacral foramina (where the SNM quadripolar electrode is typically inserted), there should be no reason to fear lead damage using loco-regional blocks. Moreover, no evidence is currently available to suggest higher risk of lead infection associated with the use of regional anesthesia. In our study, after a multidisciplinary evaluation with the anesthesiologists, all patients who needed CS underwent regional anesthesia (spinal, epidural or combined spinal-epidural) with no complications. Only one patient (patient 2) underwent general anesthesia because of an adverse drug reaction in the operating room.

### Device dysfunction after delivery

Device dysfunction after delivery may be due to displacement or damage of the quadripolar lead, extension or IPG. A few cases of IPG removal for loss of efficacy after pregnancy are reported, but device damage has never been described [6], nor has lead displacement as seen on x-ray. Wiseman [2] described two cases of total resolution of urinary symptoms after pregnancy, which no longer required SNM. The influence of hormonal changes was hypothesized in these cases to explain the clinical improvement.

In our series, after delivery and device re-activation, we observed an episode of UR that lasted 2 weeks and ended with the recurrence of spontaneous micturition (patient 5). Patient 1 experienced a worsening of urinary symptoms, but benefited from the new parameter setting. After a second pregnancy, she underwent a new worsening of symptoms that led to device removal. No technical problems were demonstrated. This patient kept the device off during both pregnancies and experienced a worsening of urinary symptoms, recurrent UTIs and UR on both occasions. We are currently unable to explain the loss of efficacy of SNM in this specific case and believe that it was unlikely to be related to the pregnancy.

Strengths of our study include the largest case series of women who carried on a pregnancy with the device switched on and the presence of a single team that performed all the implants using a standardized electrode placement technique. The close collaboration in our center among urologists, gynecologists and anesthesiologists allowed us to highlight some of the main critical issues related to the management of pregnant women under SNM. We acknowledge that this was a retrospective and descriptive data collection, the sample size was not large enough to obtain statistically significant results, and no predetermined outcomes or control group was considered.

## Conclusions

The use of SNM during pregnancy appears to be safe and did not result in morbidity for the fetus. Furthermore, risks associated with switching the device off—such as UTIs, UR and worsening of urinary symptoms—may outweigh benefits and justify maintaining the electrical stimulation throughout pregnancy. The choice of CS—when not strictly indicated for obstetric reasons—is prevalent because of urological and gynecological concern about lead damage/displacement or prolonged and painful delivery, but should be assessed case by case, considering that no significant SNM dysfunctions after vaginal delivery have yet been reported. Further studies with greater numbers of patients are required to obtain statistically significant data. In view of the scarce evidence to suggest the contrary, and in consideration of our experience to date, we recommend keeping the stimulator active during pregnancy and cooperating with gynecology colleagues for the deactivation of the device during labor and reactivation shortly post-partum.
